# To improve the communication between a community mental health team and its service users, their families and carers

**DOI:** 10.1136/bmjoq-2020-000914

**Published:** 2020-11-05

**Authors:** Priyalakshmi Chowdhury, Amir Tari, Ola Hill, Amar Shah

**Affiliations:** 1 Psychiatry, East London NHS Foundation Trust, Luton, UK; 2 East London NHS Foundation Trust, London, UK

**Keywords:** quality improvement, community mental health services, patient-centred care, mental health

## Abstract

This article describes the application of quality improvement (QI) to solve a long-standing, ongoing problem where service users or their carers felt they were not given enough information regarding diagnosis and medication during clinic assessments in a community mental health setting. Service users and carers had shared feedback that some of the information documented on clinic letters was not accurate and the service users were not given the opportunity to discuss these letters with the clinician. The aim of this QI project was to improve the communication between the community mental health team (CMHT) and service users and their carers. Wardown CMHT volunteered to take on this project. The stakeholders involved were the team manager and deputy manager, the team consultant, the team specialist registrar, team administrative manager, two carers and one service user. The project had access to QI learning and support through East London NHS Foundation Trust’s QI programme. The team organised weekly meetings to brainstorm ideas, plan tests of change to review progress and to agree on the next course of action. The outcome was an increase in service user satisfaction from 59.9% to 78% over a period of 6 months, and a reduction in complaints to zero.

## Problem

In psychiatry efficient communication is a vital part of our clinical assessments and treatment. Wardown Community Mental Health Team (CMHT) in East London NHS Foundation Trust is situated in Luton, UK. This is a daily community service which relies on effective communication between the healthcare professionals, service users and carers. The aim of this project was to increase the service user satisfaction from 66% to 78% over a period of 6 months and a reduction in complaints to zero.

Despite the importance of this everyday process, we identified long-standing issues with the service. This was reinforced by the Patient Advice and Liaison Service (PALS) data which highlighted concerns that carers and service users did not have access to adequate information regarding their diagnosis and the medicines they were prescribed.

The service users and carers felt that they should tell their story only once, instead of repeating each time they see a different member of staff whether it is a doctor, care coordinator or support worker. Other problems that were discussed included the necessity for all members of the staff to update their diaries for the forthcoming 2 weeks. This was to ensure that no service user would turn up for an appointment to be told that there was no one who could see them due to illness or holiday. Carers and service users also felt that sometimes the clinician’s report was not accurate or lacked some information and there was no avenue for feedback to be given before the letter was sent to the general physician.

Even though these problems may look relatively straight forward, it had to be tackled in a quality improvement (QI) project. This was mainly because even though there were large changes in the CMHTs in Luton under way, there needed to be a mechanism for staff and services closer to patient care to be able to make small changes that could make a real difference to people’s outcomes and experience.

## Background

Luton is a large town situated in the south of England. The 2016 population figure for Luton, as published according to the Office of National Statistics was 216 800, with some parts of the unitary authority being particularly dense in population, even when compared with London boroughs. Luton is an ethnically diverse town, with approximately 55% of the population being of Black, Asian and Minority Ethnic origin, with significant Pakistani, Bangladeshi, Indian, East European and African Caribbean communities. Due to this, Luton is increasingly being viewed as a ‘super-diverse’ community. According to the Projecting Adult Needs and Service Information estimates, between 22 872 and 23 971 people living in Luton have some sort of mental health problem and between 9559 and 10 036 have two or more psychiatric disorders. The psychiatric disorders range from the more common mood disorders, schizophrenia to the more complex post-traumatic stress disorder, attention deficit hyperactivity disorder and personality disorders.

Wardown CMHT serves approximately 48 000 people in the Luton areas of Crawley, High Town, South and Wigmore. The medical team comprises of a full-time consultant, a specialist registrar and junior doctor trainees who rotate every 4 to 6 months. The team has a lead psychologist along with clinical support workers, social workers and community psychiatric nurses. There is also an administration team along with team administrator. The whole team is managed by the team manager.

Following the data compiled from the PALS of the last 12–24 months, we designed a survey aimed at service users and carers to gain further insight into the issues facing the current service. Approximately 40 feedback responses were received from the service users. The survey revealed that 59.9% found the existing service satisfactory and 34.1% felt it was substandard. Many felt that ineffective communication resulted in confusion regarding their medicines, diagnosis which would also have long-term consequences to medication adherence.

In a wider context, we found that many of the drawbacks of the existing services were not unique to our CMHT. The impact that effective communication has on patient satisfaction has been widely reported in the literature.^1–7^ Similar projects have been undertaken both nationally and internationally, which have identified the importance of good communication between staff and service users. Saunsbury *et al*^1^ demonstrated that improved communication between phlebotomist and junior doctors was achieved by the introduction of the phlebotomy box. Sustersic *et al* reported that patient information leaflets in emergency departments in France improved the overall patient satisfaction with healthcare professionals. Priebe *et al*^3^ did a conceptual review of good communication in psychiatry. It was concluded that good communication between clinicians and service users is the basis of psychiatry treatment which also helps to achieve the clinical objectives in psychiatry.^4^ Rensburg *et al*^2^ did a similar study on effective communication and treatment adherence of patients in South Africa public sector specialist psychiatric out-patient clinic. The objectives of this study were to: explore the basic knowledge and understanding of patients’ conditions and treatment prior to the implementation of the proposed communication intervention programme; implement the communication intervention programme (‘reminder and support adherence programme’, RSAP) for an initial period of 3 months; document the experience and views of participants after the implementation of the RSAP; and compare medication and clinic attendance adherence at baseline and after the RSAP intervention. The RSAP-focussed communication interventions included weekly phone calls; free telephonic counselling; reminder SMS messages; brochures and information; SMS reminders for workshops, support groups and press notifications; an online website with information; monthly newsletters; and free support groups in different regions.^2^

It was found that effective communication not only improved the overall satisfaction but also treatment adherence. Zolnierek and Dimatteo conducted a meta-analysis of published literature (1949 to 2008), using a random effects model. They found physician communication to be significantly associated with patient adherence, with a 19% higher risk of non-adherence among patients whose physicians communicated poorly. They also reported that training physicians in communication skills resulted in substantial and significant improvements in patient adherence.^5^ Sanson-Fisher *et al*^6^ also noted that effective communication skills can and should be taught to healthcare providers as part of their training. In their qualitative review of the medical literature from 1970 to 2005, Jin *et al*^7^ reported on factors affecting therapeutic adherence from the patient’s perspective. They identified patient-centred factors, therapy-related factors, socioeconomic factors and patient-centred factors, with the latter including the patient-prescriber relationship and communication which, in particular, also had a significant effect on compliance.

### Baseline measurement

Initial data collection focussed on the PALS complaints data over a period of 12 to 24 months. A proforma was used to measure whether information had been given to the service user and how satisfied they were with the service. Our baseline data demonstrated satisfaction of 59.9% with the service, based on patient-reported experience measures.

In order to meet our project’s aim of improving experience of the service by improving communication, we used the outcome measure from the patient-reported experience measure of the percentage of service users who were likely to recommend the service

### Design

The project team consisted of the team manager and deputy manager, the team consultant, the team specialist registrar, team administrative manager, two carers and one service user. Once the project team was established, baseline data was collected and aim set for 90% of service users and carers to recommend the service to their friends and family. Initially, a form was created to give to the patients that asked whether information was given to them regarding the medicines that are prescribed along with the diagnosis.

The team attended the service user forum (working together group) which consisted of around 10 to 15 people with lived experience of using the service. From the initial meetings, it also became apparent that the service users were neither given enough information regarding their medications nor about their condition.

**Figure 1:**
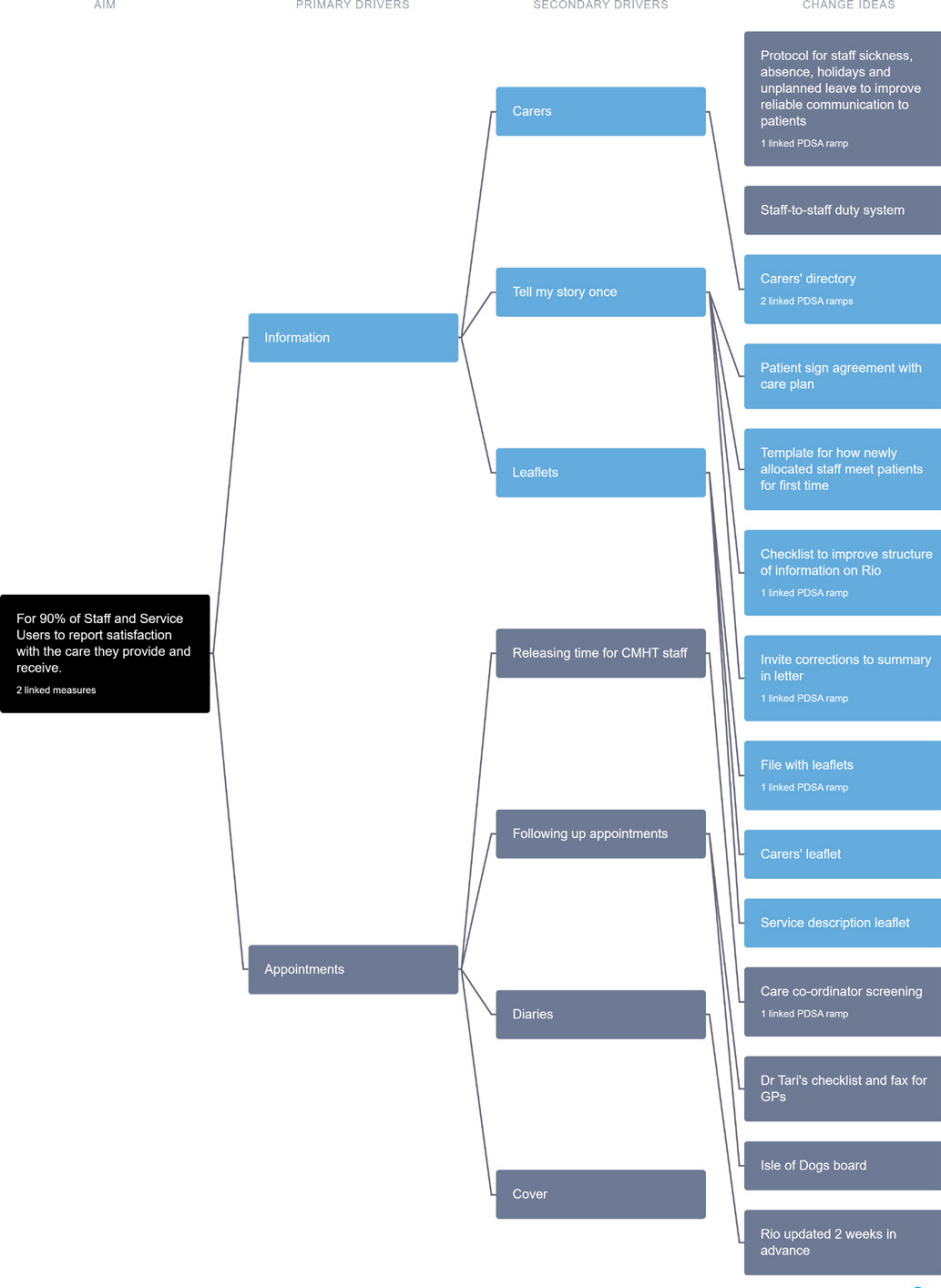
Driver diagram showing the aim, primary drivers, secondary drivers and changed ideas. CMHT, community mental health team; GP, general practitioner.

The team developed a theory of change as a driver diagram (figure 1). We used nominal group technique to develop change ideas and identified the primary and secondary drivers. We also used a technique called multi-voting to decide on which change ideas to test first.

Plan, Do, Study, Act (PDSA) cycles involved the testing of posters in clinical spaces, follow-up phone calls to service users and the amendment of clinic letters. The poster was brightly coloured and pasted on the walls of two clinic rooms of the clinicians who were involved in the project. This served as a reminder for both service users and clinicians to communicate and share information regarding the issues that has been discussed.

Following testing, we standardised the location where leaflets for both medicines and diagnosis would be kept safely in clinic rooms. After consideration of different versions of leaflets, we settled on the Trust ‘Choice of Medication’ page for leaflets which was kept in a file folder in alphabetical order for ease of access.

## Strategy

### PDSA 1

We started testing changes only in the Wardown CMHT. For continuity, we primarily focussed on this team, where issues had already been identified. In our first PDSA cycle, we introduced medication leaflets. The plan was to have medication leaflets in two clinic rooms in the clinical team’s base (Charter House) with two clinicians. The first test ran for a month. The data collected was service user feedback about the usefulness of the medication leaflets.

Medication folders were created for each clinic room. Medication leaflets were printed from the Trust ‘Choice of Medication’ page and placed in each clinic room at Charter House. Main medications were antipsychotics, antidepressants, mood stabilisers and hypnotics. All clinicians, substantive and locum, and also junior doctors were made aware that leaflets needed to be distributed to both old and new patients when a new psychotropic was started. The folder was regularly checked by the QI project team and the administrative manager. When the medication leaflets were finished, new leaflets were printed out and restored. The number of leaflets distributed was documented and records were kept. Service user feedback was obtained by follow-up phone calls to assess the usefulness of the leaflets given. Between 6 and 10 phone calls were made each week during this testing phase.

### PDSA 2

**Figure 2:**
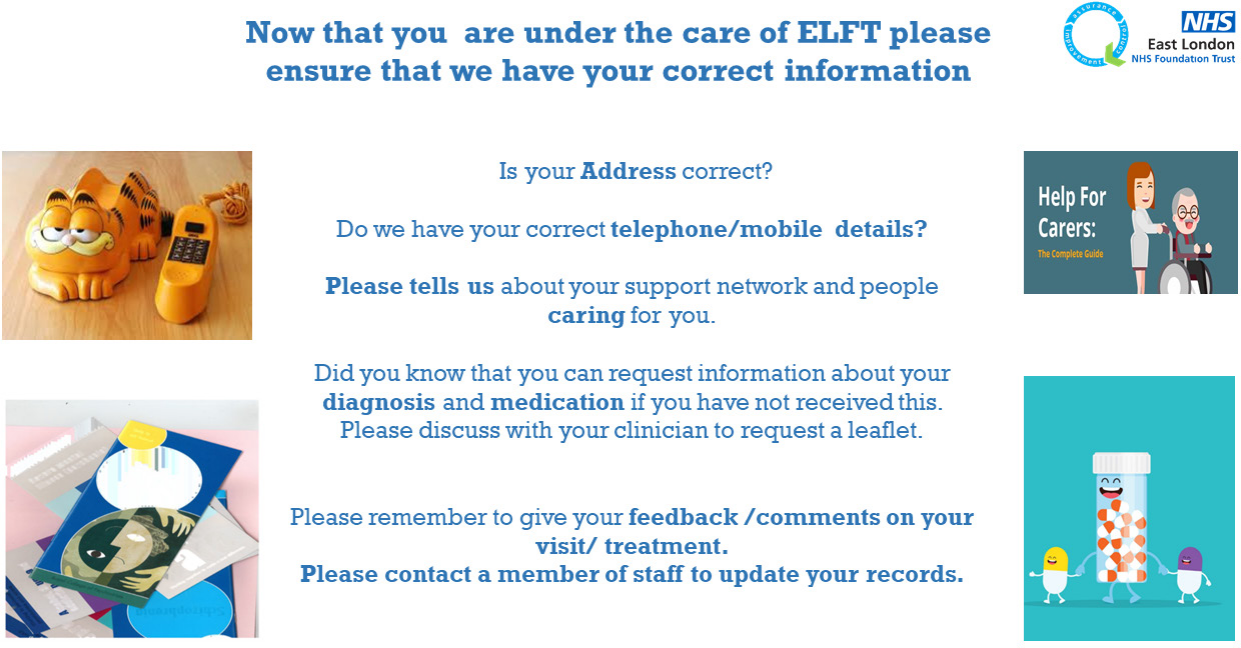
Poster set up in the clinic rooms, which served as a reminder for the clinicians, and also as a prompt for the carers and service users to ask for the patient information leaflets.

After successful completion of the first test, and based on positive feedback from service users, we scaled up the test of the medication leaflets for all the clinicians in all the rooms of the CMHT. The same pattern was followed as in the initial test. When reviewing the outcome of this second test, it was found that the medication leaflets were not given by all clinicians consistently. The project team developed a force field analysis to identify the factors supporting and restraining people from adopting this change idea. As a result, we decided to create a poster for clinicians for the clinic rooms (figure 2). This not only served as a reminder for the clinicians but also as a prompt for the carers and service users to ask for the leaflets if they did not receive any.

### PDSA 3

We decided to introduce diagnostic leaflets in the third PDSA cycle. Prior to this, meetings took place with the consultants as well as management team. It was agreed to order the Royal College of Psychiatrists diagnostic leaflets and place these alongside medication leaflets in the clinic rooms at Charter House. The QI project sponsor agreed to cover the financial cost of this. We designed a poster and placed a copy in each clinic room as a reminder for the clinicians and service users to ensure that they gave and received the leaflets, respectively. Service user feedback was obtained to help understand the impact of this change. The data demonstrated to the team that there would be a few patients who could not read the leaflet due to the severity of their mental state. However, this allowed the opportunity for the carer to take a leaflet, and the service users were aware that they could refer to the leaflet at a later point.

### PDSA 4

During our follow-up phone calls and complaints data, it was noted that service users and carers were not always happy with the content of the clinic letters. So, we expanded our trial to make some amendments to the clinic letter template by adding the following paragraph:

“If you have any urgent concerns about the contents of this letter, please do not hesitate to contact the Team Manager or the Deputy Manager, otherwise you can discuss your concerns during your next appointment.”

### PDSA 5

It was made mandatory for each member of staff to update their electronic diaries for the upcoming 2 weeks in order to become more efficient in delivering care for our service users during unexpected events such as when a member of staff was unwell. In this situation, their diary could be checked and their tasks re-allocated to another member of the team. This PDSA was monitored by the team manager and deputy team manager.

### PDSA 6

The concept of ‘Tell my story once’ was implemented as PDSA 6. A new comprehensive and holistic template was designed and implemented to record clinical information on the electronic clinical record. This information was then distributed to all the members of the Wardown community mental health service which included the nurses, social workers and care coordinators. This step was supported by the extensive education from the information technology team. The team was trained and educated where to record and look for this information in electronic records to find a patient’s complete information.

Ten months after the changes were made, a survey was done based on both quantitative and qualitative feedback from follow-up phone calls of the service users and carers, the results of which are discussed in the results section

### Measurements

The outcome measure for the project was percentage of service users and carers saying they would recommend the service. This data was collected on the routine patient experience feedback system (friends and family test).

Process measures included the number of leaflets distributed weekly, and the number of diagnostic leaflets handed out weekly.

The number of complaints and PALS enquiries was used as a balancing measure.

## Results

**Figure 3:**
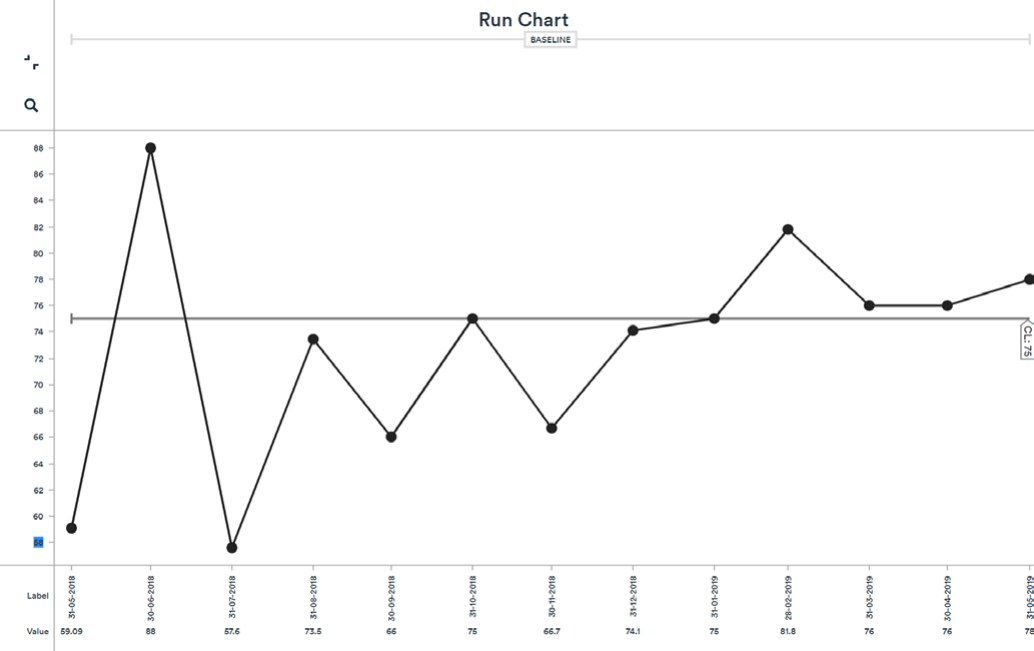
Outcome measures: Per cent of service users and carers recommending the service (run chart) lessons and limitations.

Prior to the project, in September 2018, the baseline data on the outcome measure showed that 66.6% of service users would recommend the service. After the change ideas had been tested and implemented, there was an increase to 82% of service users and carers recommending the service to friends and family (figure 3).

Over the course of the project, there were zero formal complaints about the service, and this has been maintained to the point of submitting this paper.

The data collection for the outcome measure also allowed qualitative feedback. This included comments such as ‘helpful, informative and appreciated’.

The aim of the project was to improve communication between the CMHT, and the carers and service users with the key focus of implementing a sustainable solution rather than a short-term intervention. A key lesson learnt was the importance of PDSA cycles, which helped us to ensure at each stage that the change idea was tested, adapted and optimised in our setting before full implementation across the service.

As enhancing communication was the key aim, we have learnt the essence of teamwork. This project could not have been achieved without the regular project team meetings, support from a skilled and accessible QI coach, and regular participation by the service user and the carers who were a part of this project. We also ensured that service users, carers, doctors, social worker and the team manager were involved throughout the process by attending the team meetings for regular updates on the tests being run.

The project lost one carer from the project team mid-way as she had to leave the project due to personal reasons. This resulted in destabilising the confidence of the other carer and service user. However, additional support was provided and we soon got back to our weekly regular meetings.

Some of the challenges encountered during this project were finding regular time to meet due to busy clinical commitments. However, maintaining protected time each week, and learning about QI through East London NHS Foundation Trust’s Improvement Leaders Programme at the same time as actively applying the learning to the project was extremely helpful.

## Conclusion

In summary, it was unanimously identified that lack of effective communication was a fundamental flaw in our pre-existing community mental health service. This has also been discussed extensively in literature as was also shown in a few national and international projects.

A significant improvement in communication along with improved overall satisfaction was noticed due to the changes that were implemented during this QI project, namely medication leaflets, diagnosis leaflets for service users, diary monitoring for clinical staff, and modifications to the clinic letter template.

In the next step for the QI project team, we would like to support carers within Luton. At present, this is an ongoing project and the aim will be communicated more systematically with carers, and ensure they are all aware of their rights and the support they are entitled to.
